# Using a Socioecological Framework to Understand the Career Choices of Single- and Double-Degree Nursing Students and Double-Degree Graduates

**DOI:** 10.5402/2012/748238

**Published:** 2012-07-17

**Authors:** Noelene Hickey, Linda Harrison, Jennifer Sumsion

**Affiliations:** ^1^School of Nursing and Midwifery, The University of Newcastle, Ourimbah, NSW 2258, Australia; ^2^School of Teacher Education, Charles Sturt University, Bathurst, NSW 2795, Australia

## Abstract

Untested changes in nursing education in Australia, such as the introduction of double degrees in nursing, necessitate a new research approach to study nursing career pathways. A review of the literature on past and present career choice theories demonstrates these are inadequate to gain an understanding of contemporary nursing students' career choices. With the present worldwide shortage of nurses, an understanding of career choice becomes a critical component of recruitment and retention strategies. The purpose of this paper is to demonstrate how an ecological system approach based on Bronfenbrenner's theory of human development can be used to understand and examine the influences affecting nursing students' and graduates' career development and career choices. Bronfenbrenner's socioecological model was adapted to propose a new Nursing Career Development Framework as a way of conceptualizing the career development of nursing students undertaking traditional bachelor of nursing and nontraditional double-degree nursing programs. This Framework is then applied to a study of undergraduate nurses' career decision making, using a sequential explanatory mixed method study. The paper demonstrates the relevance of this approach for addressing challenges associated with nursing recruitment, education, and career choice.

## 1. Introduction

The overall effectiveness of any healthcare system depends on a viable nursing workforce to provide optimum population health outcomes [1]. Yet that viability is under increasing threat as the recruitment and retention of nurses both in Australia and overseas reaches a crisis point [2–4]. At the time of writing, estimated shortages in Australia stand at approximately 10,000 registered nurses [5]. Furthermore, research from several comparable western countries has shown that between 30% and 61% of new graduates intend to leave nursing within their first year [6, 7]. Supply of new graduates from university bachelor of nursing (BN) programs is not able to keep up with demand [8, 9].

In 2002, in an attempt to address this problem, the Australian Federal Government increased the number of funded places for nurse education in universities [10, 11]. Many of these places became situated in new double-degree programs that combine a bachelor of nursing (BN) with another undergraduate degree. Double degrees (DDs), also known as joint, dual, or combined degrees are well established in Australia [12–14] and are slowly on the rise in Europe [15]. These DDs involving nursing are studied conjointly and can be either within a similar discipline area, for example, bachelor of nursing/bachelor of midwifery, or across two separate discipline areas, for example, the bachelor of nursing/bachelor of commerce. In 2009, over 33% of nursing students in Australia were enrolled in DDs [9, 11]. Despite their increasing popularity and rising enrolments, double degrees may bring further challenges to the recruitment of suitable people into nursing. DD nursing students' course progression and the career paths chosen by graduates are as yet unknown. 

Understanding the career decisions of graduates with a BN degree is an essential component of recruitment and retention strategies [16–18]. It is well recognised that career decisions can be shaped by students' course experiences [19, 20] as well as nonuniversity factors such as family background, family commitments, support from significant people [21, 22], and employment opportunities [23]. To date, however, limitations identified in the extant literature about nursing students' and new graduates' career decisions preclude a more in-depth understanding of the influence of these factors in the contemporary Australian context. These limitations are fourfold.

Firstly, few studies, have taken account of the constellation of contextual factors that are relevant to the decisions individuals make when choosing to enter into a career in nursing. Secondly, much previous research on students' progression through their higher education into work has been criticized for lacking a strong, explicit, theoretical basis [24, 25]. This criticism has also been made of the literature on nursing students' progression through their university studies and into work [17, 26]. Moreover, as argued by Price [18, page 268], existing career choice theories have provided limited understanding of career choice in nursing because they fail to capture the unique characteristics of upcoming generational cohorts. Thirdly, many studies in nursing education have been limited methodologically, are descriptive rather than analytical, and lack a systematic approach [18, 27]. Fourthly, despite the large and growing proportion of students enrolled in DDs including nursing in Australia, there has been almost no attention to the career decisions of DD nursing students and graduates [9, 11]. 

Given the seriousness of the nursing workforce shortages and the need to address recruitment and retention problems, the limitations of the existing literature are of particular concern. Theoretically informed approaches are needed, therefore, to identify and understand how the career decisions of single- and double-degree nursing students are influenced by a constellation of personal characteristics, experiences, development, and transitions as well as contextual factors such as those mentioned above. It is timely to identify a framework that, at a number of levels, can explain the complexities involved in the development and career choices of undergraduate nursing students. The purpose of this paper is to demonstrate how an ecological system approach based on Bronfenbrenner's [28, 29] “process-person-context-time” (PPCT) theory of human development can be used to understand and examine the influences affecting nursing students' career development and career choices. 

The paper consists of three sections. The first section examines the limitations of previous research into the educational preparation and career development of nursing students. It also includes an overview of prior development theories and their inadequacies for understanding the complexity inherent in the career choice of single and DD nursing students of today. The next section presents Bronfenbrenner's socioecological model which was adapted by the first author to provide a framework for researching nursing students' career development and career choice over time. The framework and the potential of an ecological system approach for understanding career transitions and trajectories of nursing students are explained. The third section illustrates the utility of Bronfenbrenner's framework through its application to a study of nursing career development and career choices in a sample of single- and double-degree nursing students and graduates in Australia. 

## 2. Limitations of Previous Research

### 2.1. Lack of Attention to the Many Factors Relevant to Nurses Career Decision Making

Nurses' career choices are influenced by a variety of individual, cultural, developmental, social, and environmental variables, many of which are unforeseen and unpredictable [30]). Previous studies have identified several influences on the decision to choose nursing as a career. These include family members and a desire to care for others [16, 31], exposure to healthcare environment through caring for a loved one, or prior hospital and/or work experience [31, 32]. However, these studies have taken a narrow focus either on students' characteristics, their decision, and influences at one particular time or on influences from the past. Few have included broader contextual factors such as socioeconomic status, academic achievement, demography (rural or urban background), and social ties to friends, family, and/or a lifetime partner. 

### 2.2. Lack of a Strong, Explicit Theoretical Basis for Researching Career Decision Making

The phenomenon of career choice has been studied extensively for more than a century, generating a vast array of literature on career development and career decision-making theories and models [18, 33]. The major and most frequently cited theories on career development and career choice are based on a variety of developmental, social-cognitive, personality, and person-environment fit perspectives [18]. There have been many critiques of these theories [34, 35] and of studies investigating the influences of developmental stage on career choice [36]. 

A major critique of many of the most influential theories of career development, including, for example, Personality Development and Career Choice Theory [37], Career Development Theory [38], Career Typology [39], and Social and Cognitive Career Theory [40], is that they were developed within the context of western industrialized society and therefore cannot be assumed to be universally applicable [33]. Moreover, because they were developed primarily with reference to white middle class males, mostly adolescents and up to and including the final year of schooling [41, 42], they are likely to have little relevance for university students, who have left school and taken the first steps towards a career. Gottfredson's [43, 44] Developmental Theory of Occupational Aspiration, Circumscription and Compromise endeavored to address the gender bias of previous studies and to extend previous developmental stages work by considering the influences of gender, career prestige, and interest on the compromises individuals must make when formulating career aspirations and choices. However, the focus is on what Gottfredson called the four stages of cognitive development (which included career choices) from preschool to late adolescence, rather than on adults' career decisions. 

A second major criticism is that commonly used career choice theories of the past failed to capture the challenges, complexities, and uncertainties of the 21st century workplace for the upcoming generation of nurses and lacked comprehensiveness [18]. In her extensive critical review of career choice theories and nursing, Price commented that many extant development theories were outdated and would not be able to examine “differences in developmental experiences among newer generational cohorts” nor issues “… relevant to contemporary workplaces … especially in healthcare settings” [18, page 270]. 

A further criticism, also noted by Price [18], is that past theories lend themselves to examining an individuals' career choice at one point in time and do not take into account social and environmental contextual factors and their multiple influences. Robinson and Bornholt's [25] Pathways Theory sought to address the limitation of past developmental theories. This theoretical framework was used to inform an investigation of contemporary higher education pathways and student progression in Australia [24]. Robinson mapped university students' progression pathways and outcome behaviors over time, using the categories of continuing, completing, or withdrawing from a course and transferring between courses or dropping out. Despite the emphasis of their framework on the centrality of the student, as well as the reciprocity between the students, the university context, the course context, and the changing nature of the latter two contexts over time, reasons for dropping out or transferring to another course were not revealed. Moreover, the students' perspectives were not heard. Emotions such as anxiety and uncertainty influence decisions, and the role of affect and emotions needs to be better understood and incorporated into contemporary theories of career choice [45]. 

### 2.3. Lack of a Systematic Approach to Researching Nurses Career Decision Making

Numerous studies related to career choice in nursing education literature have included a focus on reasons for choosing a career in nursing [46, 47], attrition and retention of undergraduate students [27, 48], the socialization process in nurse education and career choice [49, 50], clinical specialty area choices [20, 51, 52], and outcomes of nursing education programs for graduate attrition and retention [2, 53]. 

Many of the above studies were included in Gaynor et al.'s [17] systematic review of literature from 1996 to 2005 that quantified and examined the factors that were associated with attrition of single-degree undergraduate nursing students in preregistration programs and the retention of graduate nurses in the workforce. Of the 73 diverse studies they identified, Gaynor et al. found no high-quality studies that focused on the retention of new graduates or discussed career choices. Moreover, only four studies that met the inclusion criteria were high-quality primary analytical studies. Of the four, two studies—Deary et al. [54] from Scotland and Harvey and McMurray [55] from Australia—found the attrition rate of students at 12 months was 25–27%. The third study [56] found that nursing students from 14 colleges in the USA in 1995 were less likely to leave compared to those in 1983 (a decrease from 12% to 4%), while the fourth study [57] revealed an attrition rate of 19.3% over two years in a rural university in Australia. Overall, Gaynor et al. [17] concluded that there has not been a systematic approach to research into why students leave or continue in nursing programs and that any claims needed to be treated with caution because of methodological limitations. Gaynor et al. stated that studies “… relied on small convenience samples … and assessed intentions rather than actually measuring attrition or retention as outcomes” [17, page 28]. Moreover, as the most recent of these studies was undertaken in 2003, the findings may not be relevant to current generational cohorts and contemporary organizations and workplaces. 

Over the last two decades, there have been numerous studies to identify which area nursing students were most likely to choose to work in after graduation. In the main, these focused on nursing clinical specialty areas, such as aged care [19, 58, 59] and mental health [20, 51, 60, 61]. The majority of this research into career specialty choices and/or preferences has used longitudinal designs to identify if students' attitudes, and/or choices for popular areas (pediatrics) and unpopular areas (aged care and mental health) changed between the commencement and completion of their nursing programs. Stevens and Dulhunty [62, 63] and Stevens and Crouch's [59] seminal work in Australia identified little change in students' lack of interest in the unpopular areas. In contrast, medical-surgical and the highly technical areas of nursing became more popular over time. Later studies in Australia [20, 64] and overseas [19, 61] confirmed this finding. A number of studies identified factors that influenced these preferred career areas [20, 65], including contact with student peers, new graduate and other registered nurses, academic staff, and service users [66–68]. Hence it was a combination of curriculum theory and clinical experiences which could be either positive or negative. These studies did not extend to the postgraduate (PG) years. Further limitations included their focus on identified preferences and attitudes but not actual choices and on single-degree nursing students only. They examined specialty choices within nursing only and did not make explicit their theoretical framework. 

### 2.4. Lack of Attention to Double-Degree Students' Career Decision Making

When it comes to career aspirations, career mobility, and the wider context of work, the present generation of young people demonstrates distinct differences from their predecessors [18, 69]. They are more likely to enter university at an older age (>19 years), remain in full-time education for a longer period than their parents did, and, on graduation, to be faced with a transition to a highly differentiated skills market with an increasing range of options [10, 52, 70]. In contrast, studies of undergraduate students enrolled in double-degree programs reveal that these students tend to be school leavers (<19 years on enrolment). They also have higher tertiary entrance scores than single-degree students and are more likely to be female (60% versus 51% in single-degree programs) [13, 14, 71]. It is unknown if these characteristics are also true of nursing DD students and whether this might impact on their nursing career aspirations. The study by Russell et al. [14] was the only study found that focused on DD students' and graduates' career decision making. It was conducted in an Australian university where 1344 DD students were enrolled in 23 different DD program combinations. No theoretical framework was evident, but by using a mixed method approach the authors were able to identify the characteristics, motivations, and career transition experiences of students enrolled in DDs. They found that 75% of respondents chose a DD to improve their employment prospects and the students reported many advantages including social benefits from exposure to two sets of different people and programs. The advantages, however, were “… countered by formidable workloads, conflicting expectations and administrative difficulties” [14, page 582]. While this study is useful, there remains a paucity of studies on DD students [14, 71], and none could be found specifically on nursing students. Hence, too little is known about this group of contemporary nursing studentsand what is influencing their career choices. 

Given the cumulative effect of these limitations, there is an urgent need for a new-theoretical framework for understanding not only single-degree nursing students of today but also those in DD programs and new graduates from these programs. 

## 3. Bronfenbrenner's Socioecological Theory of Development

Urie Bronfenbrenner's socioecological theory of development [28, 29] was chosen to address the limitations cited above. This human ecology theory, also called “Development in Context” or “Ecological Systems Theory,” specifies four types of nested environmental systems which each contain roles, norms, and rules that shape development. Development is seen as a process of bidirectional influences within and between these systems. The phenomenon of development is its primary concern. The proposition is that throughout the life course a person's development occurs through ongoing reciprocal interaction between that person and the other “… persons, objects, and symbols in its immediate environment.” To be effective these interactions “… must occur on a fairly regular basis over extended periods of time” [72, page 1643]. Hence, this theory is ideal for investigating and explaining the career development of nursing DD students as they interact within the new university environment or work environment over a period of three or more years.

### 3.1. Contexts in Which Socioecological Theory Has Been Used

Bronfenbrenner's theory of development has been particularly influential in child development. Yet, his framework can be applied to different populations and is increasingly used in studies of university students. For example, Bryan and Simmons [73] examined how the family and other levels of influence played a role in the Postsecondary educational success of first-generation Appalachian American university students; Renn and Arnold [74] studied peer influences on learning and development of university students as well as how the interactions amongst the student's immediate environments create the forces of campus peer culture, and Chin and Young [75] used Bronfenbrenner's socioecological approach to understand the characteristics of beginning teachers in the Alternative Certification Programs in California.

### 3.2. Advantages of a Socioecological Theory in Nursing Research

Bronfenbrenner's socioecological model offers a developmental theory that allows for the complexity of career development to be emphasized; yet to date, it appears to have been untried in nursing career research. The starting supposition of Bronfenbrenner's model is that students' developmental pathways can vary, that development results from interactions among individuals, their activities, and their environments, and that the outcomes of professional preparation (e.g., career choice) are dependent on the development process which is always contextualized within specific environments. Bronfenbrenner's socioecological developmental theory [28, 29], therefore, provides a useful framework for understanding the transition of undergraduate nursing students through university to graduation and a career. This approach can acknowledge the complexity inherent in understanding the multiple factors that influence a student's career choice and the diversity that exists within groups as well as between groups of students. It can be applied to adult populations and as such is well suited to present day research into nursing students' career choices. It is dynamic and takes into account the wider context that can influence decisions but models this through a person-oriented approach. 

### 3.3. Components of the Socioecological Model

**Figure 1:**
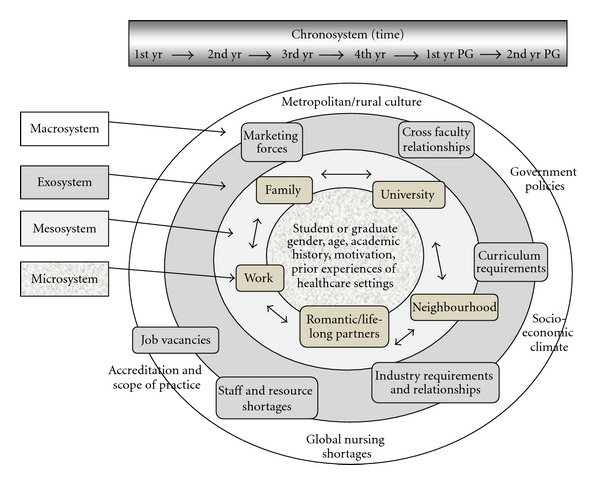
The nursing career development framework (adapted from Bronfenbrenner 1992).

Bronfenbrenner's socioecological model has four interrelating components; hence, it is often referred to as the “process-person-context-time model” (PPCT). The first component is the developmental *process* which “… involves the fused and dynamic relation of the individual and the context” [76, page xv]. The second component is the *person *(i.e., the student) who has their own biological, cognitive, emotional, and behavioral characteristics. The third component comprises the *context* where the human development occurs. It is seen as a set of nested systems—the microsystem, the mesosystem, the exosystem, and the macrosystem. The last component is *time* “… ontogenetic time, family time, and historical time—constituting the chronosystem that moderates change across the life course” [76, page xv]. The Nursing Career Development Framework presented in Figure 1 is adapted from Bronfenbrenner's model to depict the four nested systems.

### 3.4. The Person

In the context of nursing education, at the centre are the students and graduates themselves (see Figure 1, centre circle), including their personal characteristics or attributes and what they bring to their university studies and the university context, such as their age, gender, motivations, academic history, and any past experiences of healthcare settings/organizations through work or illness. In the model, the person is seen as an “active agent” who can make decisions by interpreting and manipulating the outside world about his or her own career preferences. Each nursing student may have similar experiences at university, but it is how they personally interpret these settings and the strategies they employ that contributes to his or her development.

### 3.5. The Microsystem

The microsystem “… is a pattern of activities, roles, and interpersonal relationships experienced by the developing person in a given face to face setting” containing physical and material features as well as the other persons with their own characteristics [29, page 147]. Three key settings are typically identified in depictions of socioecological contexts shaping development—family, school, and neighborhood which includes personal friends and community [77]. In young adults, romantic attachments or lifetime partners are a further microsystem for some, as is their regular place of work. In relation to nursing students, the contexts of influence depicted by boxes located in the second circle of Figure 1 are depicted as five microsystems: family, romantic partners, neighbourhood, work, and university, each of which include the persons, objects, and resources they encounter in each of these settings. The university microsystem, for example, comprises the subjects which the students are studying, the teaching and learning experiences, new campus peer groups and friendships that are formed through studying, and role models that are provided by their lecturers, and/or clinical supervisors. 

### 3.6. The Mesosystem

The mesosystem is the interrelationship between two or more microsystems in which the developing person participates. In other words it can be said that the mesosystem is a system of microsystems [78]. In relation to nursing education, it comprises the linkages and processes taking place between the settings that are important to and affect the developing student. For example, there are overlapping influences between home and university and the workplace and the university. These interrelationships are depicted by arrows between settings in the second circle in Figure 1.

### 3.7. The Exosystem

The exosystem “… consists of one or more settings that do not involve the developing person as an active participant but in which events occur that affect, or are affected by what happens in that setting” [28, page 237]. With respect to nursing education, the exosystem consists of policies and events in the wider university arena that indirectly affect the student. Depicted in the third circle of Figure 1, these include faculty curriculum requirements, industry requirements for clinical practicum, and the relationships between industry and the university. The exosystem also includes external features such as staff shortages and job vacancies in nursing, as well as marketing forces both from nursing and other professions. For example, some professional organizations such as state ambulance services have very effective marketing campaigns, and these could have a profound impact on students' ability to gain a graduate position and the promise of interesting work in a place of one's choice. In relation to DD students, cross-faculty relationships are also an exosystem influence as communication and accommodation for double-degree program scheduling across faculties can affect students' university experiences. 

### 3.8. The Macrosystem

The macrosystem refers to the overriding beliefs, values, ideology, practices, and policies that exist within a cultural group [28]. In nursing education, macrosystem influences that are outside the determination of the student or the university include urban or rural culture, the socioeconomic climate, global nursing shortages, accreditation boards, and government healthcare policies (Figure 1, outer circle). For example, in Australia the federal government's policies related to funding of universities and financial aid to students indirectly affect students' daily lives in terms of the amount of work required to pay university costs (accommodation, books) and anxiety related to finances. Who attends what university might seem to be an individual, or family-based decision; however, the factors, for example, living in a rural or a metropolitan area, which govern university-choice decisions are positioned in the macrosystem and only made manifest locally. People living in rural areas may be restricted in their choice of universities or choice of degrees due to costs, distances, or both unless they qualify for government subsidies. In Australia the other major influences in the macrosystem are the accreditation or licensing boards such as the Nurses and Midwives Board. Whilst protecting titles and professional autonomy, these boards do not allow cross-professional or multidisciplinary care even though the graduate may be licensed to practice in two disciplines, for instance, nursing and paramedics. This means that the utilization of the graduates' skills or scope of practice is restricted and they are forced into choosing one career or the other [11].

### 3.9. The Chronosystem

The chronosystem or element of time is essential to the ecological model portrayed in Figure 1. It denotes that environments change; they are not fixed identities. Historical contexts also change as do dominant discourses in society. The characteristics of the people in them and the activities of individual students are also constantly changing over time [28]. The era in which students' attend university can in part shape career choice. For example, national and global events such as the financial crisis in late 2010 are time-bound influences that can affect a student's choice of career. The chronosystem at the top of Figure 1 depicts the four years of undergraduate study and the two years following graduation as the overarching timeframe for students' and new graduates' career choices and decision making in the study outlined in the following section. 

To summarize, Bronfenbrenner's process-person-context-time theory of human development has informed the development of a framework that can give researchers a broad lens for conceptualizing and examining the career development of nursing students. It allows a modeling of how factors in the immediate as well as the wider context influence their career choices as they interact with individuals and social, educational, and clinical healthcare environments over time. The Nursing Career Development Framework presented in this paper is person centered, making it possible to build a picture of single- and double-degree nursing students and graduates and their career decisions over time. 

## 4. Applying the Nursing Career Development Framework to Investigate Nurses' Career Choice

This section illustrates how the Nursing Career Development Framework can be applied to capture the *process* of nursing students' development and career decision making during their university program and transition to work. The framework guided the development of a research design, the central goals of which were to characterize and compare single and DD BN students and graduates, identify and understand why undergraduate students enroll in a DD program, and explore the influences that affect career development and career choices during their study program and their first two years postgraduation. 

The context was a regional university in Australia that offered a traditional BN program and two nontraditional DD programs that included nursing: a bachelor of nursing/bachelor of early childhood teaching (birth to 5 years) (BN/BECT) and a bachelor of nursing/bachelor of clinical practice (Paramedic) (BN/BCP). This university in New South Wales was chosen as it was the first in Australia to have cohorts of DD graduates who were in the workforce. In order to capture the process of development over time, all students enrolled in the BN/BECT and BN/BCP double degrees and the single BN degree in all years of the program, as well as two cohorts of DD graduates, were invited to participate. The framework provided a dynamic structure that could encompass the developmental pathways of these distinct cohorts of students and graduates at different stages of their career trajectory and career decision making on enrolment, on completion, and the first and second years of work. Ethics approval was granted by this university in late 2007, and informed consent was obtained from the participating students and graduates. 

A sequential, explanatory mixed methods' design was chosen, collecting and analyzing first quantitative data and then qualitative data in consecutive phases [79, 80]. The collection of mixed data within one study strengthens the research by bringing together different but complementary data, as neither method is sufficient in itself to capture the trends in career choices [81], and provides the detailed contextual information necessary for exploring the nested systems in the framework (see Figure 1). Furthermore, in a sequential mixed method study, quantitative and qualitative data collection methods are not completely independent because one builds upon and informs the other [82]. For example, in this study analysis of a survey of the cohort of newly enrolled students generated a schedule of questions to be used in face-to-face interviews with a smaller number of DD students. 

A cross-sectional cohort study of undergraduates was designed, with data collected at regular intervals using different cohorts. The data were entered into SPSS (version 17, SPSS Inc., Chicago, IL, USA) and analysed using chi square (*χ*2) tests and analysis of variance (ANOVA). This method provided an ideal way to gather information on career decision-making processes at various stages of the students' development as they progressed through their degrees. For example, the survey of newly enrolled students was followed by a second survey of final-year students which generated additional questions for focus group interviews with DD students. 

The cross-sectional design was supplemented by the addition of a longitudinal cohort study of DD graduates; hence graduates of the DD programs comprised a third cohort. Longitudinal cohort studies can reveal factors that influence individuals and how these change, over time, and as such, are ideally suited to the socioecological approach. For example, in the longitudinal study individual telephone interviews were conducted, using some survey questions but mainly open-ended questions to record and understand career choices, career changes, and what influenced these decisions. 

The survey questions and undergraduate interviews provided information on students' preferred career choices, as well as on content areas identified in the Nursing Career Development Framework: the characteristics of the individual *persons*, their perceptions of the *contexts* of their university study or workplace that were influencing their career choices, and how these perceptions and contexts changed or were moderated over *time* that is from the beginning of their degree to the completion of their degree. As indicated in Figure 1 in the Nursing Career Development Framework, the student's personal characteristics and aspirations are central. Personal information, such as age on entry to university, gender, home postcode, and previous and present work experience and parents' socioeconomic status, along with an indication of their initial career choice preference when they first entered the undergraduate degree program, were collected in the cross-sectional surveys using a semistructured format. Open-ended questions were included to gather information about why DD students had chosen a particular nursing program (BN, BN/BECT, or BN/BCP), which discipline areas they expected to work in when they enrolled-either nursing or another discipline, whether they preferred to work in a rural or metropolitan location, and the reason(s) for their choices. Changes or confirmation in students' career preferences and the factors that may have influenced this since they began the degree were the focus of an additional set of questions that were included for DD students in their final year of study.

For the DD graduates, telephone interviews were used to collect similar information as gathered from undergraduates. Graduates were asked to comment retrospectively on: which career they envisioned when they started their DD which career and location they were presently working in and why; how long they intended to stay in their present career and location, and what was influencing those decisions. They were also asked if they intended within the foreseeable future to change professions in line with their double qualifications (e.g., from nursing to teaching or paramedics to nursing) and what factors or experiences would influence that change. 

In analyzing the quantitative and qualitative data, priority was given to identifying consistencies across groups of students and common influencing factors, for example from any of the ecological systems such as the micro-, meso-, exo-, and macrosystem. In brief the survey results demonstrated that these DD nursing students were different to SD nursing students as they were younger; were more likely to be male; and came from a higher socioeconomic background. In regards to career preferences by the final years only one–third of DD students were interested in a career in nursing and less than half of all the students from rural backgrounds intend to work as a graduate nurse in a rural location. 

Individual and focus group interviews were analysed thematically. Themes were identified at a semantic or explicit level building a picture of the students at various stages in their degree or graduate employment and the reasons for any changes in career choice. Analyses then examined how these reasons might be related to influences at the level of systems: micro-, meso-, exo-, and macrosystem. Findings showed that career choices were influenced by pay and conditions and location (rural versus metropolitan) which are features of the macrosystem, work-based practicum experiences (an exosystem influence), and family, their own work, and university experiences (microsystem influences). Additionally, students' and graduates' personal motivations such as enjoyment or preferences for interesting and challenging work were identified.

When analyzing the information provided by DD graduates interviews in the longitudinal study, the Nursing Career Development Framework provided a clear basis for understanding and explaining graduates' actual career choices, why it was that they chose that particular career, their intent to stay or leave, and the factors influencing their decisions. For example, the analysis of these data revealed factors that strongly influence the retention of registered nurses, including proximal factors acting in the microsystem such as family, work location, and work satisfaction and enjoyment, as well as indirect factors at the level of the macrosystem, for example, government policies regulating pay and work and opportunities to work as a nurse in overseas countries. Please see Hickey et al. [83] for a report on findings of this illustrative study.

In sum, the Nursing Career Development Framework provided a basis for identifying personal, interpersonal, and external influences at different levels (meso-, exo-, and macrosystems) and the degree to which these contributed to each individual's cycle of experience and career decisions and trajectories. Previous studies have not identified this type of information. 

## 5. Conclusion

In summary when nursing-funded places were increased in Australia, DDs in nursing were introduced as a new undergraduate pathway for students interested in combining a nursing qualification with another discipline. Students' motivation for choosing a DD and the educational preparation at tertiary level for two careers are situated within the particular policy context that allowed for diverse alternative pathways to a nursing career in Australia. Bronfenbrenner's socioecological theory has been applied to develop a Nursing Career Development Framework for exploring and understanding the complex issues inherent in career development and career choice for nursing students enrolled in single or double degrees in nursing. The application of this framework through a sequential explanatory mixed method design provides an illustration of a new approach for collecting and analyzing data that recognized the multiple and intersecting contexts described by Bronfenbrenner. This new approach can lead to a more thorough understanding of career development and career choice processes in undergraduate and PG nursing students, which in turn has the potential to inform strategies that can enhance recruitment and retention of future nursing professionals. Ecological approaches to studying nurses in tertiary programs in general and for double-degree programs in particular, such as the one described here, are essential in this time of nursing shortages.
